# Alterations of the Gut Microbiome Composition and Lipid Metabolic Profile in Radiation Enteritis

**DOI:** 10.3389/fcimb.2020.541178

**Published:** 2020-10-21

**Authors:** Yiyi Li, Hongmei Yan, Yaowei Zhang, Qingping Li, Lu Yu, Qianyu Li, Cuiting Liu, Yuwen Xie, Keli Chen, Feng Ye, Kai Wang, Longhua Chen, Yi Ding

**Affiliations:** ^1^ Department of Radiation Oncology, Nanfang Hospital, Southern Medical University, Guangzhou, China; ^2^ Cancer Research Institute, School of Basic Medical Sciences, Southern Medical University, Guangzhou, China; ^3^ Division of Hepatobiliopancreatic Surgery, Department of General Surgery, Nanfang Hospital, Southern Medical University, Guangzhou, China; ^4^ Medical Imaging Specialty, The First School of Clinical Medicine, Southern Medical University, Guangzhou, China; ^5^ Central Laboratory, Southern Medical University, Guangzhou, China; ^6^ Department of Pathology, Nanfang Hospital, Southern Medical University, Guangzhou, China; ^7^ HuiQiao Medical Center, Nanfang Hospital, Southern Medical University, Guangzhou, China

**Keywords:** radiation enteritis, radiotherapy—adverse effects, gut microbiome, lipid metabolism (source: MeS, HNLM), inflammation

## Abstract

Radiation enteritis (RE) is a common complication in cancer patients receiving radiotherapy. Although studies have shown the changes of this disease at clinical, pathological and other levels, the dynamic characteristics of local microbiome and metabolomics are hitherto unknown. We aimed to examine the multi-omics features of the gut microecosystem, determining the functional correlation between microbiome and lipid metabolites during RE activity. By delivering single high-dose irradiation, a RE mouse model was established. High-throughput 16S rDNA sequencing and global lipidomics analysis were performed to examine microbial and lipidomic profile changes in the gut microecosystem. Spearman correlation analysis was used to determine the functional correlation between bacteria and metabolites. Clinical samples were collected to validate the above observations. During RE activity, the intestinal inflammation of the mice was confirmed by typical signs, symptoms, imaging findings and pathological evidences. 16S datasets revealed that localized irradiation dramatically altered the gut microbial composition, resulting in a decrease ratio of *Bacteroidetes* to *Firmicutes*. Lipidomics analysis indicated the remarkable lipidomic profile changes in enteric epithelial barrier, determining that glycerophospholipids metabolism was correlated to RE progression with the highest relevance. Spearman correlation analysis identified that five bacteria-metabolite pairs showed the most significant functional correlation in RE, including *Alistipes*-PC(36:0e), *Bacteroides*-DG(18:0/20:4), *Dubosiella*-PC(35:2), *Eggerthellaceae*-PC(35:6), and *Escherichia-Shigella*-TG(18:2/18:2/20:4). These observations were partly confirmed in human specimens. Our study provided a comprehensive description of microbiota dysbiosis and lipid metabolic disorders in RE, suggesting strategies to change local microecosystem to relieve radiation injury and maintain homeostasis.

## Introduction

Radiation enteritis (RE) is a major health concern in recipients of radiation therapy (Andreyev, 2007). For abdominal, pelvic and rectal cancer patients, approximately 90% of them develop a permanent change in bowel habits after radiotherapy, and 50% have an associated reduction in quality of life (Pfaendler et al., 2015). To solve this problem, many different treatment strategies have been developed to direct toward symptom relief and management of emerging complications, but the most widely used and effective approach now is still to reduce radiotherapy dosage, which inevitably compromises therapeutic effect (Hauer-Jensen et al., 2014). Hence, the rational precision drug design based on improved understanding of the molecular basis and microecosystem changes still needs further development (Fuccio and Guido, 2013).

Human gastrointestinal microbiota, also known as gut flora or gut microbiome, is the microorganisms that live in the digestive tracts of humans. Its role in both health and disease has been the subject of extensive research (Gong et al., 2018; Lee et al., 2019; Pryor et al., 2019), establishing its involvement in human metabolism, nutrition, physiology, and immune function (McKenzie et al., 2017). Recent years, emerging evidences implicated that dysbiosis of the gut microbiota plays a crucially important role in the pathogenesis of radiation-induced intestinal injury (Crawford and Gordon, 2005; Gerassy-Vainberg et al., 2017; Sokol and Adolph, 2018). In 2017, Ming Cui et al. reported that faecal microbiota transplantation can be employed as a therapeutic to ameliorate radiation-induced toxicity and improve the prognosis of patients after radiotherapy (Cui et al., 2017). Thus, elucidating the interaction mechanisms between microbiome and host may offer potential therapeutic benefits.

In this study, we established an animal model of radiation-induced intestinal injury, then identified that localised irradiation dramatically altered the composition of the gut microbiota and resulted in lipidomic profile changes of the enteric epithelial barrier. These multi-omics alterations might well be one of the important events in the pathogenesis of RE, collaboratively driving its progression. Our data highlighted the most functionally relevant pairs between bacteria and lipid metabolites *during RE activity*, uncovering potential and novel therapeutic targets for the prevention of radiation induced intestinal injury.

## Materials and Methods

### Animal Studies

Six- to 8-week-old male C57BL/6J mice (Skwarchuk and Travis, 1998) were purchased from Guangdong Medical Laboratory Animal Center (Guangzhou, China). They were housed in the specific pathogen-free level animal facility at the Key Laboratory of Molecular Oncology Pathology, Southern Medical University. Mice were kept under standard conditions (ambient temperature 23 ± 2°C, air humidity 50 ± 10% and a 12:12 light-to-dark cycle) and continuous accessed to a standard diet and water, acclimatizing for 3-4 weeks prior to experimentation (Caruso et al., 2019). All the mice in this study were of a pure C57BL/6J genetic background and separated into groups randomly. All experiments were done in accordance with procedures approved by the Institutional Animal Care and Use Committee of Nanfang Hospital. All procedures and animal handlings were performed following the ethical guidelines for animal studies.

### Irradiation Studies

A linear accelerator (Varian Clinac 23EX Linear Accelerator, USA) at a dose rate of 500 cGy per minute was used for all experiments. Anesthetized mice were immobilized (**Figure S1**) and treated with a single dose of 18 Gy X-irradiation at a rate of 500 cGy/min for abdominal colorectal localized external radiation. After irradiation, the mice were returned to the animal facility for daily observation and treatment as described below. Sham irradiated mice were anesthetized without irradiation.

### Assessment of Radiation Injury

After irradiation, changes in appetite, water intake, bowel habits and body weight, as well as the degree of hematochezia of each mouse were monitored frequently. One week and 6 weeks after irradiation, the whole colorectal segments were fixed in 4% paraformaldehyde solution and sectioned according to standard procedures. Histopathological findings were obtained by using Masson’s trichrome stain. Blinded to the treatments, tissue sections were examined by light microscopy and scored by a gastrointestinal pathologist. A semi-quantitative histopathologic injury scoring system (**Table 1**) was used for assessment of radiation injury (Langberg et al., 1992).

**Table 1:** Histopathologic changes used in the calculation of the radiation injury score.

Index	Histologic	Score
Thickening of serosa	Slight thickening of serosa; hyperplasia of peritoneal	1
	Marked thickening of serosa	2
	Extreme thickening and fibrosis of serosa mesothelium	3
Mucosal ulcerations	Small superficial ulcerations	1
	Ulcerations involving more than half of the intestinal circumference	2
Epithelial atypia	Abnormally oriented crypts	1
	Irregular crypt regeneration with atypical epithelial cells	2
	Adenocarcinoma	3
Vascular sclerosis	Slight thickening and hyalinization of vessel wall	1
	Vessel wall double normal thickness: hyalinization and stenosis	2
	Extreme sclerosis with marked stenosis or complete occlusion; fibrinoid necrosis	3
Intestinal wall fibrosis	Submucosa double normal thickness; broadened and hyalinized collagen fibres	1
	Submucosa three to four times normal thickness; abnormal collagen fibres	2
	Massive fibrosis including muscularis	3
Lymph congestion	Dilated lymph vessels or cystic collections of lymph	1
Ileitis cystica profunda	Submucosal glandular inclusions	1
	Submucosal cysts with polypoid ekvation of the mucosa	2
	Large cysts extending into the muscularis	3

### Immunohistochemistry

Colorectal tissue sections were examined by a modified IHC method. Antigen retrieval was achieved by boiling tissue sections for 7 min in 0.01 mol/L sodium citrate, pH 6. Sections were blocked for 1 h in 1% BSA at room temperature and incubated in anti-PCNA (#13110, Cell Signaling Technology), anti-phospho-histone H3 (Ser10) (#53348, Cell Signaling Technology) or anti-γH2AX (ab2893, abcam) antibodies at 4°C overnight. After washing, samples were incubated with horseradish peroxidase (HRP)-conjugated anti-rabbit IgG, and then by Biotin-XX-Tyramide amplification (Invitrogen), and streptavidin-HRP. Stained sections were visualized using 3,30-diaminebenzidine tetrahydrochloride (DAB) and counterstained with hematoxylin. IHC staining without primary antibody was used as a negative control.

### Quantitative Real-Time Polymerase Chain Reaction

Colorectal tissue samples were immediately homogenized with liquid nitrogen in a mortar after dissection. Total RNA was isolated using Trizol Reagent (Takara Bio) according to the manufacturer’s instructions. The RNA was reverse transcribed to generate cDNA using the cDNA Synthesis Kit (Takara Bio). Quantitative real-time PCR was performed using SYBR Green PCR Kit (Takara Bio) and 7500 Fast Real-Time PCR System (AB Applied Biosystems). Relative expressions of IL-1β (F: 5’-CAACCAACAAGTGATATTCTCCATG-3’; R: 5’-GATCCACACTCTCCAGCTGCA-3’), IL-6 (F: 5’-TGAGAAAAGAGTTGTGCAATGGC-3’; R: 5’-GCATCCATCATTTCTTTGTATCTCTGG-3’), TNF-α (F: 5’-CCACGCTCTTCTGTCTACTGAACTT-3’; R: 5’-GAGAAGATGATCTGAGTGTGAGGGTCT-3’), TGF-β (F: 5’-CAACTATTGCTTCAGCTCCACAGA-3’; R: 5’-TTCCAACCCAGGTCCTTCCTA-3’), ATM (F: 5’-GATCTGCTCATTTGCTGCCG-3’; R: 5’-GTGTGGTGGCTGATACATTTGAT-3’), ATR (F: 5’-ACTTTTACGGATTGCAGCAACT-3’; R: 5’-CCATTCCATAACCTCACCCAC-3’), DNA-PK (F: 5’-AAACCTGTTCCGAGCTTTTCTG-3’; R: 5’-TCTCAATCTGAGGACGAATTGC-3’), Chk1 (F: 5’-TGTCGCTGTGCTTGGAGTC-3’; R: 5’-AAGTTTGCACCAAATCCCAGT-3’), Chk2 (F: 5’-TGACAGTGCTTCCTGTTCACA-3’; R: 5’-GAGCTGGACGAACCCTGATA-3’), p53 (F: 5’-GTCACAGCACATGACGGAGG-3’; R: 5’-TCTTCCAGATGCTCGGGATAC-3’), Mdm2 (F: 5’-GGATCTTGACGATGGCGTAAG-3’; R: 5’-AGGCTGTAATCTTCCGAGTCC-3’), MDC1 (F: 5’-GTGGCTCCTTGGGGTATAGTG-3’; R: 5’-GGGCTTCGACCAACTACATTC-3’), Cdc25A (F: 5’-ACAGCAGTCTACAGAGAATGGG-3’; R: 5’-GATGAGGTGAAAGGTGTCTTGG-3’), Cdc25C (F: 5’-ATGTCTACAGGACCTATCCCAC-3’; R: 5’-ACCTAAAACTGGGTGCTGAAAC-3’), RAD51 (F: 5’-AAGTTTTGGTCCACAGCCTATTT-3’; R: 5’-CGGTGCATAAGCAACAGCC-3’), Mre11 (F: 5’-CCCTGACAATCCTAAGGTGACC-3’; R: 5’-CGTAGTCGGATAAGAGGCTTCC-3’), BRCA2 (F: 5’-TCTTTCTCCGAGTATCAGGAAGT-3’; R: 5’-GCAGAAGTGTCAGTGAGAGTG-3’), PARP1 (F: 5’-GGCAGCCTGATGTTGAGGT-3’; R: 5’-GCGTACTCCGCTAAAAAGTCAC-3’), BRCA1 (F: 5’-CTGCCGTCCAAATTCAAGAAGT-3’; R: 5’-CTTGTGCTTCCCTGTAGGCT-3’) and 53BP1 (F: 5’-ATGGACCCTACTGGAAGTCAAT-3’; R: 5’-GAACCTGGCTTTCAGGCTGAG-3’) were calculated using the ΔΔCt method. β-actin (F: 5’-CTCCCTGGAGAAGAGCTACGAGC-3’; R: 5’-CCAGGAAGGAAGGCTGGAAGAG-3’) was used as internal control.

### Bioluminescence Imaging

For imaging of reactive oxygen species generation in abdomen, bioluminescence images in living mice were captured using In-Vivo FX PRO at 1 min after injection with L-012 solution (Invitrogen) at a dose of 25mg/kg (Wirtz et al., 2017). Region of Interest (ROI) Tool was used to measure the fluorescent intensity. Data were collected as photons per second per centimeter squared using Bruker MI SE.

### Magnetic Resonance Imaging

MRI for *in vivo* mouse imaging was undertaken on a 7.0 T animal MRI scanner (PharmaScan70/16 US, Bruker Biospin MRI GmbH) with a mouse body volume coil-40mm. RF coil with small diameter immobilized the mice body and restricted its movement. Mice were anaesthetized with 1.5%–2% isoflurane during MRI procedures. Respiratory signals were monitored with a physiological monitoring system (SA Instruments). A rapid acquisition with relaxation enhancement pulse sequence as T2-weighted with the following parameters was used: field of view = 30 × 20 mm, matrix size = 256 × 256; slice thickness = 1 mm; average = 8, TR=2465 ms, TE=25 ms. Images were acquired, covering the volume of abdomen from the small intestine to the anus. Total 30 coronal slices, time for each scan was approximately 10 min. Images were viewed and processed using RadiAnt DICOM Viewer imaging software.

### Sample Collection

For this study, the formed faecal of mice were freshly collected from colorectum 1 week and 6 weeks after irradiation. From December 2018 to August 2019, we collected six faecal samples and 12 serum samples from patients with colorectal carcinoma who underwent neoadjuvant radiotherapy. All patients signed informed consent. This study was approved by the Ethics Committee of Nanfang Hospital, Southern Medical University. All these samples were stored at -80°C until use for bacterial diversity analysis or global lipidomics analysis.

### Bacterial Diversity Analysis

Stool samples were freshly collected and stored at -80°C until use. DNA was extracted from the stool using E.Z.N.A. ^®^ Soil DNA Kit (Omega Bio-tek, Norcross, GA, USA). The V3-V4 hypervariable regions of the bacteria 16S rRNA gene were amplified with primers 338F (5’-ACTCCTACGGGAGGCAGCAG-3’) and 806R (5’-GGACTACHVGGGTWTCTAAT-3’) by Thermal Cycler PCR System (GeneAmp 9700, ABI, USA). The resulting PCR products were extracted from 2% agarose gels and further purified using the AxyPrep DNA Gel Extraction Kit (Axygen Biosciences, Union City, CA, USA), then quantified using QuantiFluor™-ST (Promega, USA) according to the manufacturer’s protocol. Samples of the same concentration were taken for Illumina sequencing. Purified amplicons were pooled in equimolar and paired-end sequenced (2 × 300) on Illumina Sequencing Platform (Illumina, San Diego, USA) according to the standard protocols by Majorbio Bio-Pharm Technology Co. Ltd (Shanghai, China). Raw fastq files were quality-filtered by Trimmomatic and merged by FLASH according to standard procedures. Operational taxonomic units (OTUs) were clustered with 97% similarity cutoff using UPARSE software (v7.1, http://drive5.com/uparse/). Representative sequence for each OTU was screened for further annotation. For each representative sequence, the Silva (SSU123) Database was used based on RDP classifier (http://rdp.cme.msu.edu/) algorithm to annotate taxonomic information. Bacteria abundance of OTUs was calculated as the proportion of their clean tags in total clean tags.

### Global Lipidomics Analysis

Global lipidomics screening was performed by ultra-high-performance liquid chromatography coupled with mass spectrometry (UHPLC-MS) analysis. Lipids extraction of mice colorectal tissues was performed by using a modified Bligh and Dyer procedure: mice colorectal tissues were collected and washed with buffer (25 mM Tris–HCl, pH 7.4, 150 mM NaCl). Lipids from tissue homogenates (2 mg protein per sample) and serum samples were extracted with chloroform/methanol/water (2/2/1.8, volumetric ratios). The lipid extracts were dried under N_2_ gas stream and reconstituted in 1:1 chloroform/methanol (Han et al., 2005; Breil et al., 2017). Each sample was analyzed by a Thermo Fisher Scientific Vanquish Flex UHPLC equipped with Thermo Fisher Scientific Orbitrap Fusion Tribrid High Resolution Mass Spectrometer (Thermo Fisher Scientific; Waltham, MA, USA). Briefly, 5μL was injected and chromatographic separation was carried out at 40°C on an Acclaim column C30 (150 × 2.1mm, 3.0 µm, Thermo Fisher Scientific; Waltham, MA, USA). The mobile phase A was: 10mM HCOONH4, 0.1% formic acid, acetonitrile (ACN): H2O= 60: 40; the mobile phase B was: 10mM HCOONH4, 0.1% formic acid, isopropanol (IPA): ACN= 90: 10; flow rate: 0.3mL/min. Electrospray ionization source was used with both positive and negative ion modes. Mass scan was performed as following settings: orbitrap resolution: 60000; scan range: m/z 200-2000; positive ion: 3500V; negative ion: 3000V; radiofrequency lens: 60%; automatic gain control target: 2.0e5; maximum injection time: 100 ms; polarity: positive. Identification of lipid molecular species was performed using Lipid Search Software 4.1.16 based on MS/MS (Thermo Fisher Scientific; Waltham, MA, USA). Result of individual sample was normalized to protein concentration. The UPLC–MS/MS data of lipidomics were subjected to MetaboAnalyst (https://www.metaboanalyst.ca/) for heatmap analysis (Xia et al., 2015), principal component analysis (PCA) and other analysis.

### Relational Networks Analysis

The Niche-specific relational networks and specific “bacterium-metabolite” relational networks were visualized using Cytoscape software version 3.7.1 (https://cytoscape.org/). Spearman correlation analysis was used for both of them. The Niche-specific relational networks mainly reflect the species correlation at genus levels under different environmental conditions. The species in the top 50 of the total abundance at genus level were selected, and the correlation coefficients among them were calculated to reflect the correlation between species at genus level. The correlation coefficients ≥ 0.8, FDR ≤ 0.05 were selected to be shown. The size of the nodes in the figure indicates the genus abundance, and different colors represents different phylum of genus; the colors of the lines indicates positive and negative correlation, red indicates positive correlation, and green indicates negative correlation. The more lines, the closer relationships between one genus and others. The specific “bacterium-metabolite” relational networks show all the significant “bacteria genus-lipid metabolite” pairs selected based on FDR ≤ 0.05. Lines in green and red respectively indicate positive and negative correlations.

### Statistical Analysis

GraphPad prism software was used to perform statistical analysis. Data are expressed as the mean ± standard error of triplicates. Each experiment was repeated at least 3 times. Student’s paired and unpaired t test, one-way ANOVA, two-way ANOVA, Wilcoxon-Rank test and Kruskal-Wallis H test were used to assess differences with *P<0.05, **P<0.01, ***P<0.001, ****P<0.0001 or *q value<0.05, **q value<0.01, ***q value<0.001, ****q value<0.0001. Multiple comparison was adjusted by Bonferroni correction.

## Results

### Establish a Well-Characterized Animal Model of Radiation-Induced Intestinal Injury

**Figure 1:**
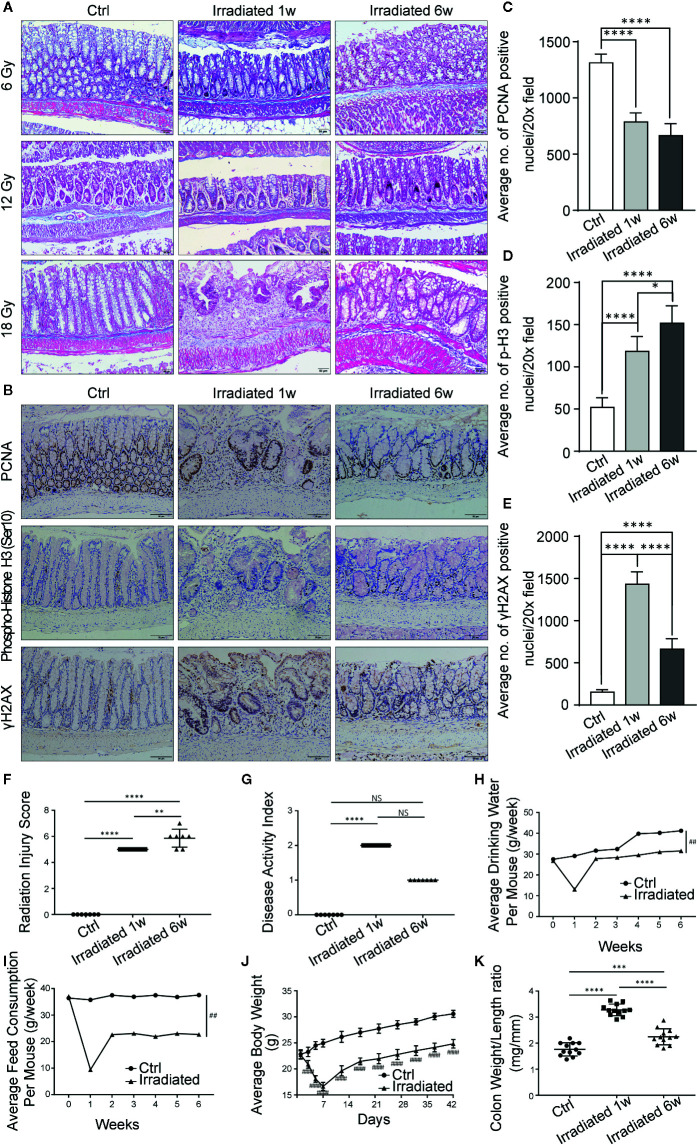
Histopathologic and systemic symptom changes of RE mice. **(A)** Masson’s trichrome stain of colorectum at 1 week and 6 weeks post-irradiation. **(B)** Immunohistochemical staining of colorectum for PCNA, phospho-histone H3 (Ser10) and γH2AX at 1 week and 6 weeks postirradiation. **(C–E)** Quantification of PCNA, phospho-histone H3 (Ser10) and γH2AX positive nuclei in colorectral cells. **(F–K)** Evaluation of RE severity by using **(F)** radiation injury score, **(G)** disease activity index, **(H)** water consumption, **(I)** feed consumption, **(J)** body weight and **(K)** weight/length ratio of colorectum. Significance levels are indicated as follows:****q < 0.0001, ***q < 0.001, **q < 0.01, *q < 0.05; NS q > 0.05; ^####^p < 0.0001,^##^p < 0.01.

Radiation enteritis (RE) is most common in people receiving *radiotherapy* for cancer in the abdomen and pelvic areas (Stacey and Green, 2014). Advances in this field that rely on human studies have been slow and seriously restricted by practical and logistic reasons (Andreyev et al., 2010). To better define the pathophysiology of RE, we established a mouse model by delivering single high-dose X-irradiation to abdominal localized field with a linear accelerator (**Figure S1**). Mice were irradiated with the doses of 6Gy, 12Gy or 18Gy, then 18Gy was finally defined as the optimal dose to induce acute RE based on typical histopathologic changes and radiation injury score (**Table 1**) after 1 week of irradiation (**Figures 1A, F**), without leading to lethal acute gastrointestinal syndrome. At that time, 18Gy also caused typical symptoms of acute RE, including poor appetite, low water intake, severe diarrhea and continuous weight loss (**Figures 1H–J**).

It has been reported that pathological features of chronic RE usually occur after 6 weeks of irradiation (Gerassy-Vainberg et al., 2017; Sokol and Adolph, 2018), so we chose this time point to observe radiation-induced chronic intestinal injury. Typical histopathologic changes and radiation injury score (**Table 1**) confirmed the chronic phase of the disease (**Figures 1A, F**). Meanwhile, the aforementioned acute symptoms also alleviated in these mice. They displayed slowly restored appetite, water intake and body weight, as well as gradually disappeared diarrhea (**Figures 1H–J**).

DNA double-strand breaks (DSBs) are considered as the most lethal form of DNA damage and a primary cause of cell death and are induced by ionizing radiation (IR) during radiotherapy (Huang and Zhou, 2020). Immunohistochemistry was used to detect the proliferation and mitosis of cells in the colorectal samples in our model. Comparing with unirradiated samples, the number of proliferating cell nuclear antigen (PCNA) positive cells is significantly reduced in irradiated samples (**Figures 1B, C**). Besides, the phosphorylation of serine 10 in histone H3 [phospho-histone H3 (Ser10)] stain indicating mitosis shows significantly higher staining in irradiated samples, especially in the chronic recovery phase, reflecting the repair of radiation damage and the regeneration of intestinal epithelial cells as time goes on (**Figures 1B, D**). Moreover, we further examined the phosphorylation of histone H2AX on serine 139 (γH2AX). Results showed that colorectal epithelium was severely damaged with high γH2AX expression after radiation compared with unirradiated epithelium, especially in the acute phase, directly displaying radiation induced DSBs (**Figures 1B, E**).

Based on weight loss, stool consistency and the degree of intestinal bleeding, the disease activity index (**Table 2**) was further evaluated (Wirtz et al., 2017). The score results were highly consistent with the actual symptoms at different phases of RE (**Figure 1G**). In addition, abnormal shortening and thickening of colorectum are thought to be positively correlated with enteritis activity (Jin et al., 2017). Autopsy results revealed significant changes in colorectum length and in the ratio of colorectum weight to unit length with the disease progression (**Figure 1K**). Thus, a well-characterized animal model of radiation-induced intestinal injury is preliminarily established.

**Table 2:** Scoring system for calculating a disease activity index based on stool consistency and the degree of intestinal bleeding.

Score	Stool consistency	Blood
0	Normal	Negative hemocult
1	Soft but still formed	Negative hemocult
2	Soft	Positive hemocult
3	Very soft; wet	Blood traces in stool visible
4	Watery diarrhea	Gross rectal bleeding

### Verification of Radiation Enteritis in Molecular and Imageological Levels

**Figure 2:**
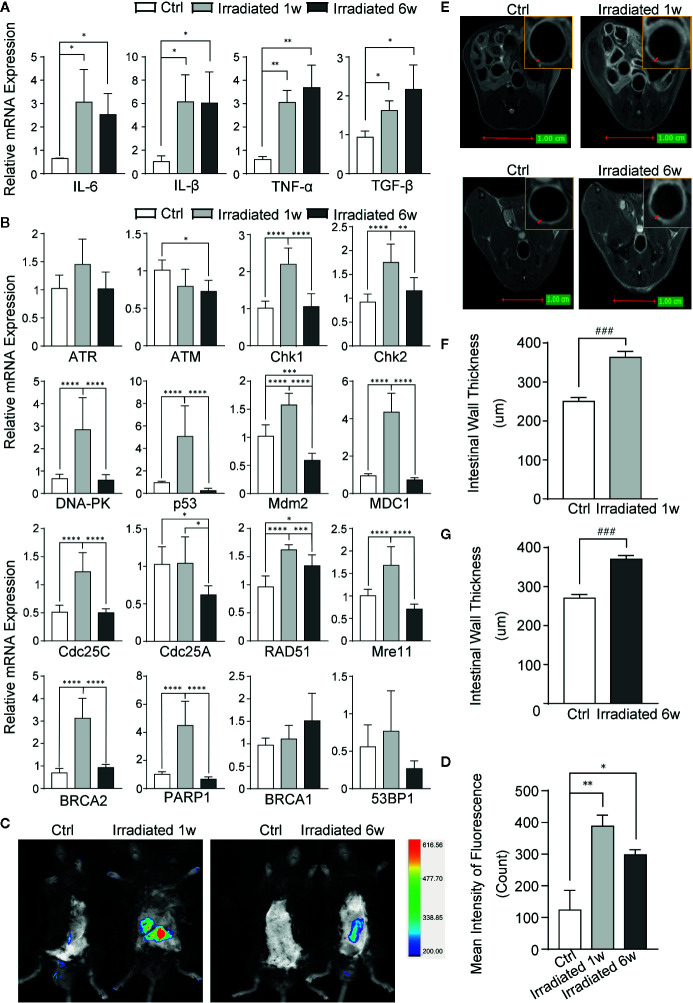
Evaluation of RE by biomarkers and imageological examinations in mice. **(A)** The expression of classical inflammatory cytokines. **(B)** The expression of genes involved in DNA damage and repair in response to radiation. **(C)** Detection of ROS by using Luminol-based chemiluminescent probe L-012 and In-Vivo FX PRO *in vivo* imaging system. **(D)** Quantification of the fluorescence signal intensity of ROS at 1 week and 6 weeks post-irradiation. **(E)** MRI for radiation-induced regional inflammation at 1 week and 6 weeks post-irradiation. **(F, G)** Intestinal wall thickness in MRI, as indicated by red arrows. Significance levels are indicated as follows: ****q < 0.0001, ***q < 0.001, **q < 0.01, *q < 0.05; ^###^p < 0.001.

Radiation-induced intestinal inflammation was further verified in both molecular and imageological levels. Intestinal specimens were examined for the expression of classical inflammatory cytokines. As shown, IL-1β, IL-6, TNF-α, and TGF-β are significantly overexpressed in RE specimens compared with control, although there is no statistical significance between acute and chronic phases (**Figure 2A**).

Radiation induced DSBs can trigger a series of cellular DNA damage responses (DDRs), which are controlled by three related kinases: ATM, ATR, and DNA-PK (Blackford and Jackson, 2017). Although there is no remarkable change in ATM and ATR in colorectal tissues of mice, significant increase of DNA-PK and several genes associated with DNA damage, such as Chk1, Chk2, p53, Mdm2, MDC1, Cdc25A, and Cdc25C, is observed in the tissues in 1 week post-radiation as compared with unirradiated samples (**Figure 2B**). Several DNA damage repair genes, such as RAD51, Mre11, BRCA2, PARP1, BRCA1, and 53BP1, are also increased in irradiated colorectal samples with statistical significance in most of them (**Figure 2B**). These results further verified the nuclear chromatin changes in our model during radiation enteritis process.

Luminol-based chemiluminescent probe L-012 is widely used *in vitro* and *in vivo* to detect reactive oxygen species (ROS) (Zielonka et al., 2013), which can truly reflect the severity of radiation damage. After 1 week or 6 weeks of irradiation, these mice were intraperitoneally injected with L-012 solution at a dose of 25 mg/kg. Then, bioluminescent animals’ images were obtained by using In-Vivo FX PRO *in vivo* imaging system. The images showed dramatically increased ROS in the intestine of RE mice, especially in acute phase, indicating different levels of radiation damage during RE process (**Figures 2C, D**). Furthermore, magnetic resonance imaging (MRI) was also used to exam radiation-induced regional inflammation. As shown in T2 weighted images (T2WI), the light ring represents the mucosa, submucosa, muscular layer and serosa of the intestine (**Figure 2E**). Compared with healthy mice, the intestinal wall thickness is significantly increased in both acute and chronic RE mice (**Figures 2F, G**). This finding is in line with histological changes, which show the presence of edema and infiltration of inflammatory cells in RE mice (**Figure 1A**).

These data strongly proved that our RE animal model exhibits an amazing symptomatological, pathological and imageological similarity to Homo sapiens, providing an effective model system for further studies.

### Dysbiosis of the Gut Microbiota During Radiation Enteritis Process

**Figure 3:**
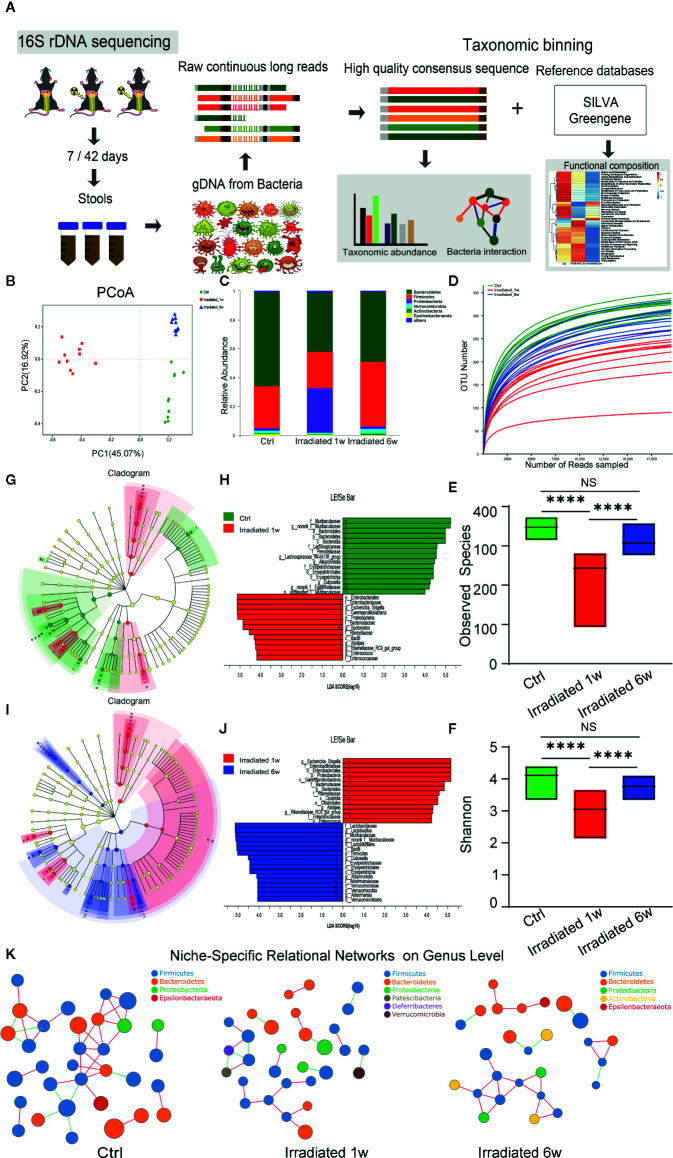
Dynamic characteristics of the gut microbial profile in RE mice. **(A)** Workflow of the 16S rDNA sequencing and data analysis. **(B)** PCoA scores plot based on OTU level. **(C)** Relative abundance of the top 10 dominant bacteria at the phylum level. **(D)** *OUT* number, **(E)** observed species number and **(F)** Shannon diversity index of intestinal bacteria among groups. **(G, J)** LEfSe analysis for genomic features of gut microbiota expressed by taxonomic cladograms **(G, I)** and liner discriminant analysis **(H, J)** based on OTU abundance. Circles irradiated from the inside to the outside represent the classification levels from phylum to genus. **(K)** Niche-specific relational networks in the genus level in irradiated and unirradiated groups. The size of the nodes in the figure indicates the genus abundance, and different colors represents different phylum of genus; the colors of the lines indicates positive and negative correlation, red indicates positive correlation, and green indicates negative correlation. The more lines, the closer relationships between one genus and others. Significance levels are indicated as follows: ****q < 0.0001; NS q > 0.05.

Preliminary studies have shown that gut microbiota composition is associated with the changing inflammatory intestinal milieu and radiation-induced tissue damage. To evaluate the impact of radiation treatment on the gut bacterial population, faecal samples of irradiated and control mice at two postradiation time points were analyzed by 16S rDNA sequencing (**Figure 3A**). Principal Coordinates Analysis (PCoA) was used to display microbiome patterns of these samples based on their similarity. As shown, each group is well separated with 45.07% and 16.92% variation explained by PC1 and PC2, respectively (**Figure 3B**). Comparing with unirradiated group, a significant shift of microbial composition is noted in irradiated group, suggesting that localized radiation dramatically altered gut microbial composition.

Taxonomic assignment and relative abundance of each gut bacterial component at the phylum level were further analyzed, and significant changes among groups were found (**Figure 3C**). As the two dominant components of human and mice gut bacteria, a trend for a lower frequency of *Bacteroidetes* and a higher frequency of *Firmicutes* is observed in irradiated groups compared with unirradiated groups (**Figure 3C**). It is well known that some *Bacteroidetes* act as probiotic bacteria in the host gut. These kinds of bacteria produce short-chain fatty acids such as butyrate, which helps to reduce inflammation and maintain the gut wall (Oliphant and Allen-Vercoe, 2019). Therefore, the decreased ratio of *Bacteroidetes* to *Firmicutes* (B/F ratio) may partly reflect the typical features of gut microbiome dysbiosis and intestinal injury during RE process (Zhong et al., 2019). Besides, the *operational taxonomic unit (OTU)* number, observed species number and Shannon diversity index of intestinal bacteria remarkably decrease in irradiated mice (**Figures 3D–F**), especially in 1 week post-radiation, indicating a trend of gradual microbial response to radiation exposure and the consequent restorations in the intestinal environment.

To further assess radiation-induced B/F ratio changes and other alterations during RE process, LEfSe analysis was used to identify genomic features of gut microbiota under different biological conditions. As shown in the figures, genus *Bacteroides*, genus *Alistipes*, genus *Alloprevotella*, genus *Rikenellaceae*, and genus *Muribaculaceae* in the *Bacteroidetes* phylum, genus *Enterococcus*, genus *Lachnospiraceae*, and genus *Dubosiella* in the *Firmicutes* phylum, genus *Eggerthellaceae* in the *Actinobacteria* phylum, as well as genus *Escherichia-Shigella* in the *Proteobacteria* phylum, all show significant abundance changes in 1 week postradiation as compared with unirradiated group (LDA score>4, p<0.05) (**Figures 3G, H**). Interestingly, this effect is largely reversed in chronic phase of the disease (LDA score>4, p<0.05) with the exception of genus *Eggerthellaceae* in the *Actinobacteria* phylum (**Figures 3I, J**). Interestingly, as probiotics, the genus *Lactobacillus* in the *Firmicutes* phylum and the genus *Akkermansia* in the *Verrucomicrobia* phylum show a significantly increased abundance in 6 weeks postradiation (LDA score>4, p<0.05), which is supposed to be involved in the recovery of the disease (**Figures 3I, J**). It is reported that gut microbiota can form niche-specific relational networks, which may partly reflect the disease states (Nakatsu et al., 2015). Based on our analysis, we did find these networks that show the microbiota-related niche-specificity at different stages of RE (**Figure 3K**).

Hence, under the big background of B/F ratio changes, the dynamic characteristics of the microbial profile of these two dominant bacteriophyta and other compositions largely reflects the dysbiosis of gut flora during RE from acute phase to chronic phase, that may help to further illuminate the pathogenesis of RE and provide potential microbiological therapeutic targets to maintain homeostasis.

### Dysbacteriosis-Related Host Metabolic Changes With the Disease Progression

**Figure 4:**
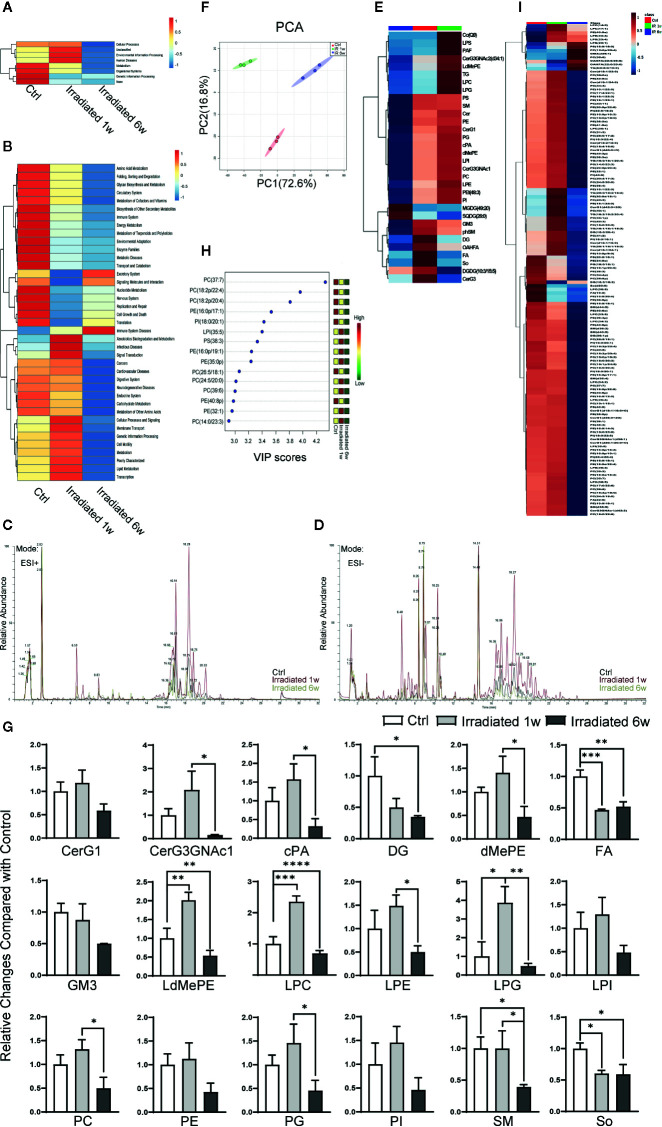
Global lipidomics profile alterations of intestinal epithelial tissues in RE mice. **(A, B)** Functional capabilities of microbial communities predicted by PICRUSt based on 16S datasets. **(C, D)** Representative MS spectra in both **(C)** ESI positive and **(D)** ESI negative ion modes for lipidomic profile in irradiated and unirradiated groups. **(E)** Clustering patterns of lipid metabolite types in irradiated and unirradiated groups displayed by hierarchical clustering heatmap. **(F)** PCA scores plot based on lipid metabolites. **(G)** 18 significantly changed lipid categories in irradiated groups compared with control. **(H, I)** The top 15 and a total of 138 lipid metabolites significantly related to RE were identified by OPLS-DA. Significance levels are indicated as follows: ****q < 0.0001, ***q < 0.001, **q < 0.01, *q < 0.05.

A large number of studies have found that intestinal bacteria make important contributions to intestinal epithelial tissues metabolism (Jiang et al., 2017; Liu et al., 2018; Chittim et al., 2019; Oliphant and Allen-Vercoe, 2019). To investigate the effect of radiation-induced gut microbiota dysbiosis on host metabolism, PICRUSt was utilized to predict the functional capabilities of microbial communities based on 16S datasets (Langille et al., 2013). As expect, RE-related dysbacteriosis is predicted to be tightly related to the host metabolic changes (**Figure 4A**). Further predictions indicated that multiple metabolic processes, including glycometabolism, amino acid metabolism, lipid metabolism, nucleotide metabolism, vitamins metabolism, and others, may all change as a consequence of RE-related dysbacteriosis (**Figure 4B**). As is known, lipid bilayer is a universal component of all cell membranes, playing an important role to maintain intestinal epithelium integrity (Trimble and Grinstein, 2015). The destruction of intestinal epithelial barrier is acritical factor affecting the progression of RE. Therefore, intestinal epithelial lipid metabolism was further characterized in our animal model.

Global lipidomics analysis using liquid chromatography–mass spectrometry (UHPLC-MS) was performed for intestinal tissue specimens. As shown, lipidomic profile of acute RE, chronic RE, and control group all shows representative MS spectra in both ESI positive and ESI negative ion modes (**Figures 4C, D**). By employing MarkerLynx XS, we calculated data matrices and exported them to SIMCA-P+ software, then obtained 1676 variables in ESI+ mode (**Figure 4C**) and 730 variables in ESI− mode (**Figure 4D**). After taking the union of these two sets, merging isomers and removing the variables with missing data, a total of 1204 substances were identified. Hierarchical clustering heatmap, which can identify relatively homogeneous groups of variables based on detected molecular features, displayed entirely different clustering patterns in acute RE, chronic RE and control group (**Figure 4E**). Similarly, PCA indicated an excellent separation with 72.60% and 16.80% variation explained by PC1 and PC2 among groups based on lipid metabolite fingerprints (**Figure 4F**). Subsequent analysis further showed that 18 categories of lipids significantly change in acute or chronic RE group compared with control, including glucosylceramide (CerG1), CerG3GNac1, cyclic phosphatidic acid (cPA), diglyceride (DG), phosphatidylethanolamine (dMePE), dimethyl fatty acid (FA), ganglioside GM3, lysodimethyiphosphatidylethanoiamine (LdMePE), lysophosphatidylcholine (LPC), lysophosphatidylethanolamine (LPE), lysophosphoglycerol (LPG), lysophosphatidylinositol (LPI), phosphatidylcholine (PC), phosphatidylethanolamine (PE), phosphatidylglycerol (PG), phosphatidylinositol (PI), sphingomyelin (SM), and sphingosine (So) (**Figure 4G**). However, changes in the total amount of these categories may not accurately reflect the correlation of specific substances with the disease (Gault et al., 2010; Amati, 2012).

To further defined potential diagnostic markers of RE, orthogonal partial least squares discriminant analysis (OPLS-DA) was performed. A total of 138 lipid metabolites are identified to be significantly related to RE (VIP≥1.0, p<0.05) (**Figure 4I**), and the top 15 most relevant metabolites can be divided into seven phosphatidylcholines [PC(37:7), PC(18:2p/22:4), PC(18:2p/20:4), PC(26:5/18:1), PC(24:5/20:0), PC(39:6), PC(14:0/23:3)], 5 phosphatidylethanolamines [PE(16:0p/17:1), PE(16:0p/19:1), PE(35:0p), PE(40:8p), PE(32:1)], 1 phosphatidylinositol [PI(18:0/20:1)], 1 lysophosphatidylinositol [LPI(35:5)] and 1 phosphatidylserine [PS(38:3)] (**Figure 4H**). Interestingly, except for LPI(35:5), all these above metabolites belong to glycerophospholipids (phosphoglycerides), which are found in highest amounts in the membranes of all cells and are responsible for the membrane being a bilayer (Powers and Trent, 2019). As shown, comparing with control, PS(38:3), PE(16:0p/19:1), PE(35:0p), PC(24:5/20:0), PC(39:6), PE(32:1), and PC(14:0/23:3) temporarily increase in 1 week postradiation, then significantly decrease below baseline values in 6 weeks postradiation (**Figure 4H**). Meanwhile, PC(37:7), PC(18:2p/22:4), PC(18:2p/20:4), PE(16:0p/17:1), PI(18:0/20:1), PC(26:5/18:1), and PE(40:8p) display a continued decreasing trend with the disease progression (**Figure 4H**). Thus, we believe that the characteristic lipidomic profile reflect the lipid metabolic changes of host at different phases of the disease. As a promising diagnostic marker, glycerophospholipids metabolism may become effective therapeutic target to maintain intestinal epithelium integrity during RE process.

### Functional Correlation Analysis Between Gut Microbiome and Lipid Metabolites

In gut microecosystem, gut microbiome and intestinal epithelial lipid metabolites play crucially important roles to maintain the stability of local microenvironment, but the functional correlation between them are less well understood (Wang et al., 2016). To explore this, pairwise Spearman correlation analysis was conducted on 12 bacteria and 138 lipid metabolites, which have all been proved the significant differences in acute or chronic RE groups. As a result, 56 out of 1656 bacteria-metabolite pairs (3.38%) showed significant functional correlation, including 40 pairs (71.43%) existing positive correlation and 16 pairs (28.57%) existing negative correlation (**Supplementary Table S1**).

**Figure 5:**
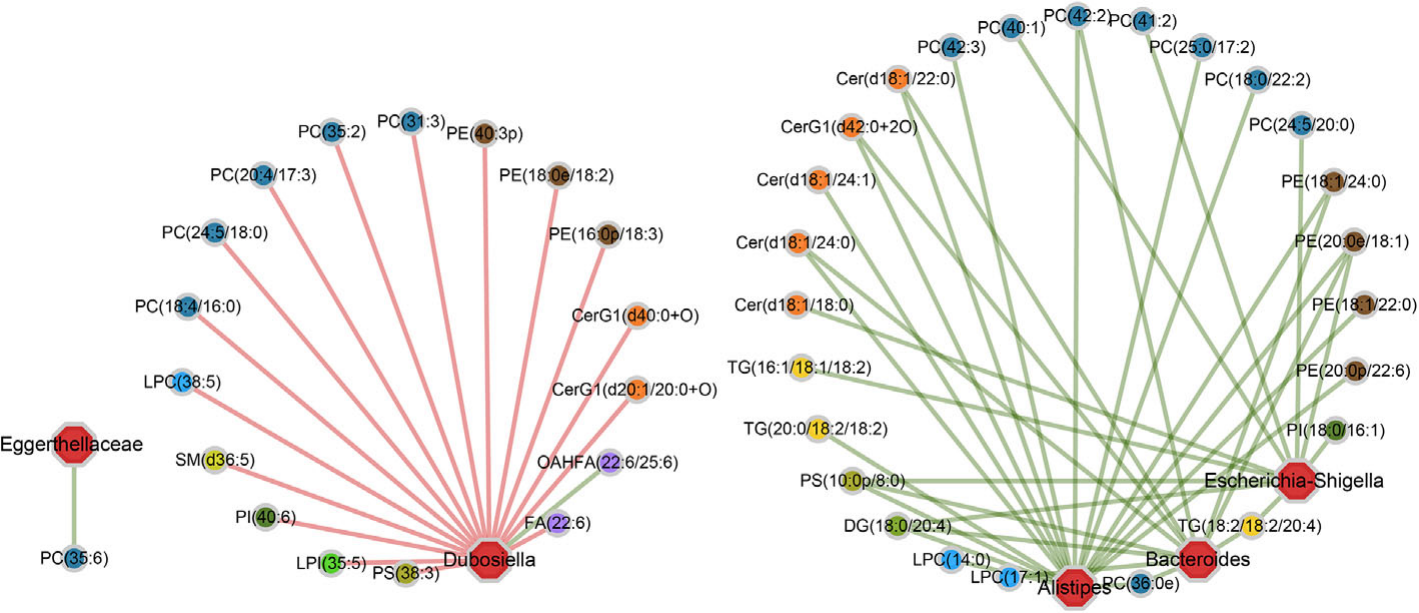
The specific “bacterium-metabolite” relational networks show all the significant “bacteria genus-lipid metabolite” pairs selected based on FDR ≤ 0.05. Lines in green and red respectively indicate positive and negative correlations.

As shown in the correlation networks, genus *Dubosiella* and genus *Alistipes* are the top two bacteria that are significantly related to most of the differential lipid metabolites, indicating their great contributions to the perturbations of lipid metabolism during RE process (**Figure 5**). More specifically, comparing with control group, the *Dubosiella* abundance decreased in acute RE group and recovered in chronic RE group, accompanying with significant changes in 17 lipid metabolites (**Figure 5**). Meanwhile, the increased abundance of *Alistipes* in acute RE group and recovered in chronic RE group significantly related to the changes of 17 lipids metabolites (**Figure 5**). Furthermore, the alterations of 10 and 11 metabolites are respectively associated with the elevated abundance of genus *Escherichia-Shigella* and genus *Bacteroides* in acute RE group as compared with control (**Figure 5**). Genus *Eggerthellaceae* is associated with only one lipid metabolite (**Figure 5**). Surprisingly, the abundance change of genus *Akkermansia* and *Lactobacillus*, the probiotic associated with anti-inflammation and multiple metabolic processes, are not related with any differential lipid metabolite in function (**Figure 5**) (Tu et al., 2018). Similarly, genus *Alloprevotella*, genus *Rikenellaceae*, genus *Muribaculaceae*, genus *Enterococcus* and genus *Lachnospiraceae* are also not related with any differential lipid metabolite. In addition, based on Spearman’s rank correlation coefficient (r value), we further analyzed the most relevant one-to-one correspondences between the above five bacteria and their corresponding metabolites. The result turned out that *Alistipes*-PC(36:0e) (r=+0.97), *Bacteroides*-DG(18:0/20:4) (r=+0.99), *Dubosiella*-PC(35:2) (r=-0.97), *Eggerthellaceae*-PC(35:6) (r=+0.96) and *Escherichia-Shigella*-TG(18:2/18:2/20:4) (r=+0.99) show the most significant functional correlation in the progression of RE.

Taken together, these dynamic correlation networks generally reflected the functional correlation between local microbiome and lipid metabolites from acute to chronic phase of RE, giving us new insights into the pathogenesis of the disease. Importantly, the identification of the most relevant bacteria-lipid metabolite pairs provided a novel biomarker panel which may collaboratively predict the severity and prognosis of RE, uncovering potential diagnostic and therapeutic targets at different phases of radiation-induced intestinal injury.

### Clinical Validation of Microbial and Lipidomic Profile Changes in Human Specimens

**Figure 6:**
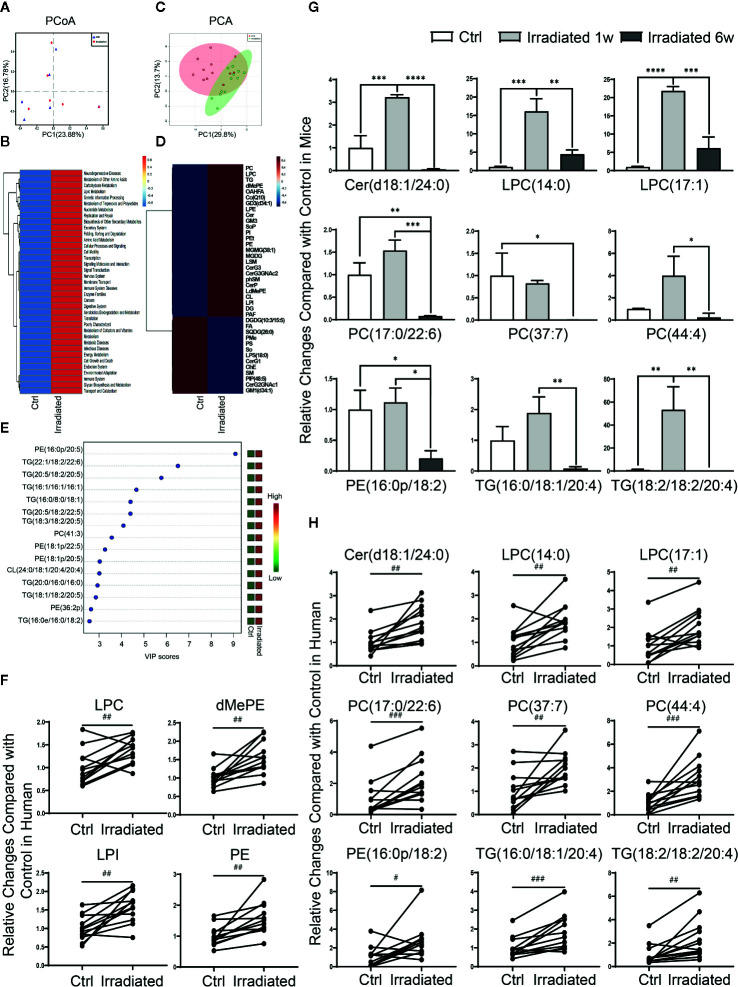
Characteristic gut microbial and lipidomic profile changes in RE patients. **(A)** PCoA scores plot based on OTU level. **(B)** Functional capabilities of microbial communities predicted by PICRUSt based on 16S datasets. **(C)** PCA scores plot based on lipid metabolites. **(D)** Clustering patterns of serum lipid metabolite types in irradiated and unirradiated patients displayed by hierarchical clustering heatmap. **(E)** The top 15 serum lipid metabolites significantly related to RE were identified by OPLS-DA. **(F–H)** Four lipid categories and nine lipid metabolites show the consistent variation trends in both human and mouse in RE. Significance levels are indicated as follows: ****q < 0.0001, ***q < 0.001, **q < 0.01, *q < 0.05; ^###^p < 0.001, ^##^p < 0.01, ^#^p < 0.05.

Although the above animal model provides a good microbiological and metabolic therapeutic research platform and contribute solid experimental basis for preclinical study, human specimens are still needed to validate current conclusions. Hence, feces specimens of rectal cancer patients before and after neoadjuvant radiotherapy were collected and analyzed by 16S rDNA sequencing. PCoA indicated that neoadjuvant radiotherapy partly altered these RE patients gut microbial composition, despite the most significant differences were existed among individuals rather than experimental groups (**Figure 6A**) (He et al., 2018). In addition, consistent with our animal study, PICRUSt functional prediction shows that radiotherapy-related dysbacteriosis may be closely associated with multiple metabolic processes changes including lipid metabolism alterations in these patients with RE (**Figure 6B**).

Due to the restriction on practical reason, human serum specimens were collected to investigate lipidomic profile changes instead of intestinal tissue specimens in these patients before and after neoadjuvant radiotherapy. Although PCA didn’t display an ideal separation explained by PC1 and PC2 between groups (**Figure 6C**), lipidomic profile revealed that the levels of most lipid metabolites are significantly altered in RE patients after radiotherapy (**Figure 6D**), including 13 lipid categories and 167 lipid metabolites with statistic difference. Further analysis showed that PE(16:0p/20:5), TG(22:1/18:2/22:6), TG(20:5/18:2/20:5), TG(16:1/16:1/16:1), TG(16:0/8:0/18:1), TG(20:5/18:2/22:5), TG(18:3/18:2/20:5), PC(41:3), PE(18:1p/22:5), PE(18:1p/20:5), CL(24:0/18:1/20:4/20:4), TG(20:0/16:0/16:0), TG(18:1/18:2/20:5), PE(36:2p) and TG(16:0e/16:0/18:2) are the top 15 most relevant metabolites with human RE (**Figure 6E**), which may take important responsibility for the disease progression. Moreover, after taking the union of human and mice sets, four lipid categories (LPC, dMePE, LPI and PE) as well as nine lipid metabolites [Cer(d18:1/24:0), LPC(14:0), LPC(17:1), PC(17:0/22:6), PC(37:7), PC(44:4), PE(16:0p/18:2), TG(16:0/18:1/20:4), and TG(18:2/18:2/20:4)] show the consistent variation trends in both human and mouse species (**Figures 6F-H**).

Our clinical data not only validate the animal experimental results to some extent, but also uncover characteristic microbial and lipidomic profile changes in RE patients. In consideration of the limitation of sample size, the difference of sampling location, the heterogeneity among individuals as well as the presence or absence of cancer, further researches is urgently needed to provide a more precise and comprehensive description of multi-omics disorders in RE from mouse to human species.

## Discussion

In this study, we first established an animal model of radiation-induced intestinal injury, and validated its similarity to Homo sapiens at biomarker, histopathologic, imageological, and systemic symptom levels. We then demonstrated that localised irradiation dramatically altered the composition of the gut microbiota, resulting in the lipidomic profile changes of the enteric epithelial barrier. We also identified the most significantly relevant pairs between bacteria and lipid metabolites that might well be responsible for RE activity. These multi-omics alterations and correlations partly reflected the dynamic features of the gut microecosystem during RE process, implying a potential pathogenesis in driving the RE progression at new perspectives.

Epidemiological and clinical studies on symbiotic microbiota have experienced a renaissance in recent years (Chen et al., 2017; Liao et al., 2018; Liu et al., 2018). Compositional and functional changes in commensal microbiota are thought to be involved in the pathogenesis of many diseases (Kahrstrom et al., 2016). In the case of radiotherapy patients, several studies have indicated that gut microbiota plays a crucially important role in the nosogenesis of radiation-induced intestinal injury. In 2017, Giuseppe L. Banna et al. reported that use of *Lactobacillus rhamnosus* could prevent the occurrence of diarrhea in patients receiving radiotherapy (Banna et al., 2017). In 2017, Ming Cui et al. reported that faecal microbiota transplantation could be employed as a therapeutic to ameliorate radiation-induced toxicity and improve the prognosis of patients after radiotherapy (Cui et al., 2017). Obviously, based on current trends, understanding how the enteric microbiota affects health and disease requires a paradigm shift from focusing on individual pathogens to an ecological approach that considers the community as a whole (Coyte and Rakoff-Nahoum, 2019). Therefore, we used high-throughput 16S rDNA sequencing to examine the alterations of the gut microbiome *composition* after radiation exposure. Our results showed that the abundance changes of 12 kinds of bacteria—*Alloprevotella*, *Alistipes*, *Akkermansia*, *Bacteroides*, *Dubosiella*, *Eggerthellaceae*, *Enterococcus*, *Escherichia-Shigella*, *Lactobacillus*, *Lachnospiraceae*, *Muribaculaceae* and *Rikenellaceae*—may be closely related to RE activity. According to their specific changing tendencies at different phases, we proposed that the increased abundance of *Alistipes*, *Bacteroides*, *Enterococcus*, *Escherichia-Shigella*, and *Rikenellaceae* makes great contributions to acute RE-related gut microbiota dysbiosis. Meanwhile, the temporarily decreased abundance of *Alloprevotella, Dubosiella*, *Lachnospiraceae* and *Muribaculaceae* reverses in chronic phase, which is probably beneficial to the recovery of acute RE and the maintenance of intestinal homeostasis. Interestingly, as probiotics, both *Akkermansia* and *Lactobacillus* show a significantly increased abundance in chronic recovery phase of the disease, implying their potential role in reversing radiation intestinal damage. It should be pointed out that several studies in both human and mice have already proved *Lactobacillus* as a probiotic bacteria to reduce radiation intestinal injury (Ciorba et al., 2012; Banna et al., 2017), but *Akkermansia* has not been studied yet in this aspect.

Currently, single omics study is insufficient to systematically explain the occurrence and development of the disease, conducting integrated multi-omics studies are increasingly becoming an irresistible trend. Recent years, it is well established that gut microbiome and their metabolites can widely regulate host metabolism, but the interactions of the molecular interface among them are highly dynamic and complex, which are still less well understood (Saw et al., 2017; McGrail et al., 2018; Lloyd-Price et al., 2019). Based on 16S datasets, we predicted that multiple metabolic processes of the host, including intestinal epithelial lipid metabolism, may all change as a consequence of RE-related dysbacteriosis. As known, lipid bilayer plays an important role to maintain intestinal epithelium integrity. The destruction of intestinal epithelial barrier is a critical factor affecting the progression of RE. Hence, we performed global lipidomics analysis for intestinal tissue specimens and used Spearman correlation analysis to identify the functional correlation between gut microbiome and lipid metabolites during RE progression. Our results indicated that glycerophospholipids metabolism is most significantly related to RE activity; the abundance change of *Dubosiella* and *Alistipes* makes great contributions to the perturbations of lipid metabolism in the disease process. Furthermore, five bacteria-lipid metabolite pairs—*Alistipes*-PC(36:0e), *Bacteroides*-DG(18:0/20:4), *Dubosiella*-PC(35:2), *Eggerthellaceae*-PC(35:6), and *Escherichia-Shigella*-TG(18:2/18:2/20:4)—show the most significant functional correlation in the progression of RE. Noticeably, it’s the first time presentation of the crosstalk among radiation exposure, micropopulation and host metabolism at the multi-omics level in different phases of RE. Their functional correlation gives us new insights into the potential pathogenesis of the disease.

Inevitably, there are three main limitations in our research. First, by employing high-throughput 16S rDNA sequencing, we assessed and compared the structure of microbial communities at a high phylogenetic resolution, but a view on the functional capabilities of gut microbiota gets lost with the data restriction. Although PICRUSt was utilized to predict the functional profiles of the community based on 16S datasets, whole metagenome shotgun sequencing still needs to be performed (Mallick et al., 2019), which can provide entire genetic information of environmental samples and present precise profiles of community structure and function (Yachida et al., 2019). Next, we used global lipidomics analysis and OPLS-DA to identify significantly changed lipid metabolites in RE mice, aiming to find potential diagnostic markers of the disease. However, for some powerful metabolites, slight quantity alterations are sufficient to trigger remarkable metabolic changes; for some extremely trace amount of metabolites, they may perform important functions but cannot be detected at all based on the current untargeted lipidomics approach (Bismuth et al., 2008). Hence, identification of functionally important lipid metabolic pathways and performing targeted lipidomics analysis for corresponding key metabolites are urgently required in the future. The last but not least, although multi-omics correlation analysis (MOCA) largely reflected the dynamic characteristics of RE at entire microecosystem level, it just explained the correlation but not causation between multi-omics changes and the disease. In consideration of the actuality that the interpretation and application of MOCA does not go far enough to date, further studies should be conducted to explore the expression profile changes and the functional alterations of the most central genes that are existed in both host and microbiome samples.

In light of the pivotal role of multi-omics changes in RE progression, the current report provides scientific evidences for the first time that characteristic microbial and lipidomic profiles may collaboratively predict the severity and prognosis of RE, uncovering potential strategies to change local microecosystem to relieve radiation injury and maintain homeostasis.

## Conclusion

The results indicate that microbiota dysbiosis and lipid metabolic disorders concurrently occur in the gut with radiation injury, it may be closely related to the occurrence and development of RE. Five bacteria-lipid metabolite pairs show the most significant functional correlation in RE progression, which provide experimental evidence and research direction for exploring the crosstalk among radiation exposure, micropopulation and host metabolism at entire microecosystem level.

## Data Availability Statement

The datasets generated and analyzed for this study can be found in the NCBI SRA database: https://www.ncbi.nlm.nih.gov/bioproject/PRJNA665135.

## Ethics Statement

The animal study was reviewed and approved by Animal Care and Use Committee of Nanfang Hospital.

## Author Contributions

HY and YL contributed conception and design of the study and wrote the first draft of the manuscript. YZ and KW organized the database. QPL and LY performed the statistical analysis. QYL, CL, YX, KC, and FY revised sections of the manuscript. LC and YD contributed to manuscript revision, read, and approved the submitted version. All authors contributed to the article and approved the submitted version.

## Funding

This work is supported by National Natural Science Foundation of China (No. 81803063, No. 81872470 and No. 81672992), Natural Science Foundation of Guangdong Province (No. 2018030310297), Guangdong Natural Science Funds for Distinguished Young Scholar (No. 2015A030306015), Guangdong Program for Support of Top-notch Young Professionals (No. 2015TQ01R279), Outstanding Youths Development Scheme of Nanfang Hospital, Southern Medical University (No. 2018J009), Foundation of President of Nanfang Hospital, Southern Medical University (No. 2017C016), and Provincial College Students’ Innovation and Entrepreneurship training Program, Southern Medical University (No. S201912121083 and No. S201912121079).

## Conflict of Interest

The authors declare that the research was conducted in the absence of any commercial or financial relationships that could be construed as a potential conflict of interest.
