# Supplementary figures and images for: Alterations of the Gut Microbiome Composition and Lipid Metabolic Profile in Radiation Enteritis
Source: Front Cell Infect Microbiol. 2020 Oct 21;10:541178. doi: 10.3389/fcimb.2020.541178 (PMC7609817; doi:10.3389/fcimb.2020.541178)

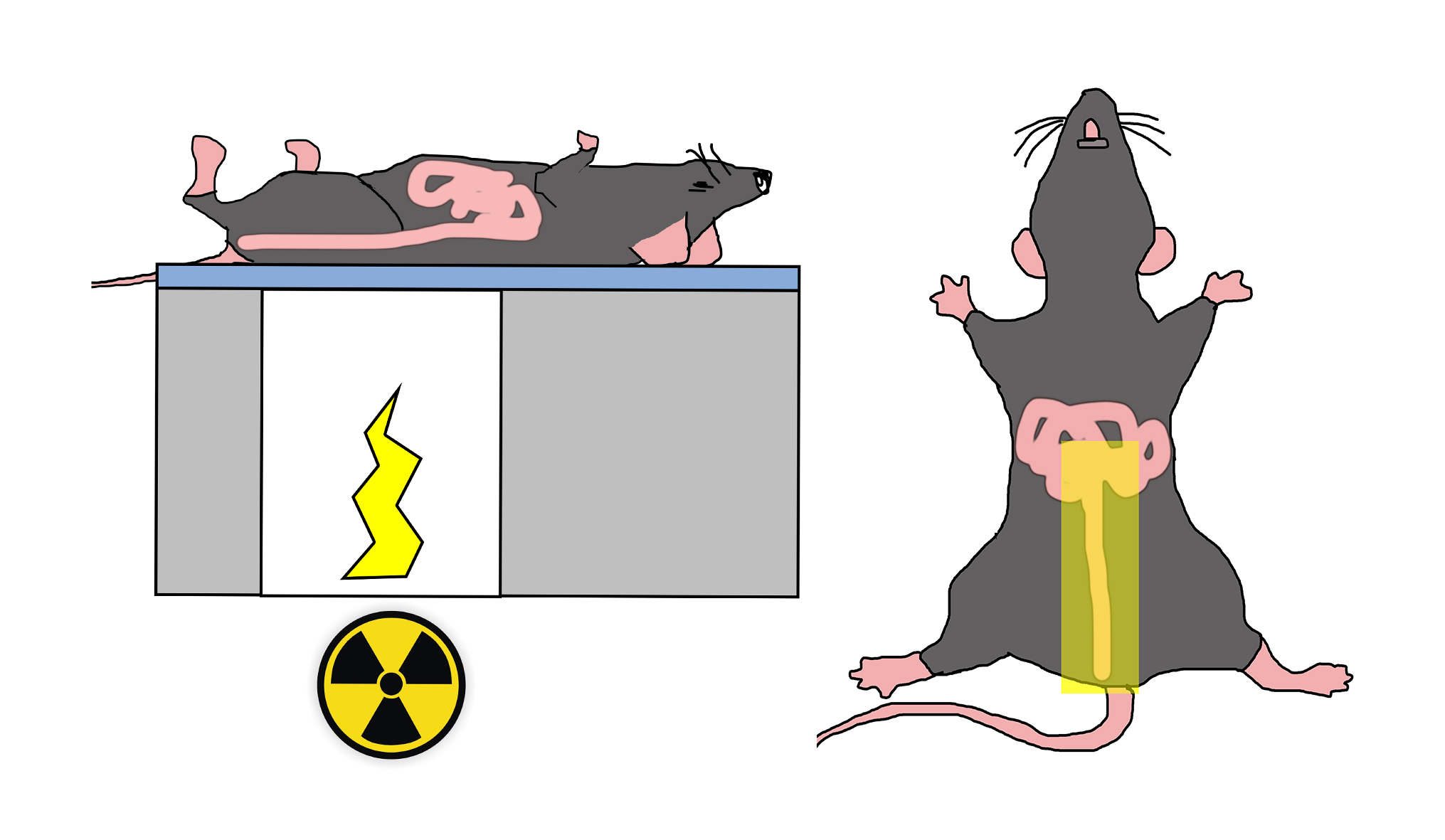

Supplement: Supplementary file 2 [file Image_1.jpg]
